# Book Notices, Etc.

**Published:** 1877-09-20

**Authors:** 


### BOOK NOTICES, ETC.

*Naphey's Therapeutics.*—Already the edition of this work which was published at the commencement of the present year is entirely exhausted. No higher testimony to its worth could be given. It recommends itself at once to every physician who sees it.

A new edition (the fifth) is in active preparation. The editor has been assisted by several very competent gentlemen in special departments, and the work has received a most thorough revision and very large additions. Indeed, so extensive are the latter, that the two parts into which the work is divided, viz: 1, Medical Therapeutics, and 2, Surgical Therapeutics, will each make a volume by itself, quite as large as that which embraced both divisions in the last edition (about 600 pages). They will be printed on handsome tinted paper, in the best style, and each part, as wholly independent, will be sold separately if desired.

A classified list of works on Surgery and the colateral sciences, published by Lindsay & Blakeston, Philadelehia,—many of them at greatly reduced prices. Descriptive catalogues sent free upon application.

Also by the same House, Physician's Visiting Lists, for 1878. The first book of the kind published in this country and a great convenience to the practitioner. Price $1.00 to $3.00, according to style, etc.

*Nurse and Patient and Camp Cure*, by S. Weir Mitchell, M.D., author of "Wear and Tear," "Fat and Blood" etc., Philadelphia, J. B. Lippencott & Co., 1877.

*The Respiratory Brace.*—A new appliance devised for the relief of orthopnœa, by Geo. F. French, M.D., Portland, Maine.

